# Data of ant community compositions and functional traits responding to land-use change at the local scale

**DOI:** 10.3897/BDJ.10.e85119

**Published:** 2022-07-06

**Authors:** Xiang Zhang, Zhi-xing Lu, Nian-nian Zhang, You-qing Chen

**Affiliations:** 1 Institute of Highland Forest Science, Chinese Academy of Forestry, Kunming, China Institute of Highland Forest Science, Chinese Academy of Forestry Kunming China; 2 Guizhou Academy of Forestry, Guiyang, China Guizhou Academy of Forestry Guiyang China

**Keywords:** land-use change, disturbances, ant assemblages, trait distribution, functional diversity

## Abstract

**Aim**: Off-reserve conservation is a major contributor to China biodiversity conservation efforts, biodiversity conservation being achieved within afforestation and low-intensity agriculture in fragmented landscapes. Functional trait is more strongly related to ecological processes than taxonomic diversity and reflects ecosystem functioning and species responses to environmental changes. In this study, we selected five habitats that differ in degree of disturbance to explore the effects of land use on ant community compositions, traits distributions and functional diversity change. We assessed how habitat disturbance affects the ant community compositions and traits distributions and asked if ant functional diversity respond to disturbance at the local scale?

**Location**: Lüchun County, Yunnan Province, southwest China.

**Methods**: Pitfall traps were used to survey ant communities. Additionally, we measured four ant morphological traits (eyes diameter, distance between eyes, femur length of the hind-leg and Weber’s length) to assess the functional traits distributions and functional diversity. Shade plot of ant relative abundance was used to explore species distribution amongst different habitats. Kernel density plot was used to explore ant traits distribution patterns amongst different habitats. Non-metric multi-dimensional scaling ordination, based on ant Weber's length, was used to explore the ant traits compositions amongst different habitats. The fourth corner model was used to evaluate the association between ant traits and environmental variables. The FR_ic_, RaoQ and FE_ve_ indices were selected as three complementary measures of the multivariate functional traits space and functional redundancy of different habitats.

**Results**: We collected 14258 ants, representing 89 species, 40 genera and seven subfamilies. *Aphaenogasterschurri* and *Tetramoriumciliatum* were the common species of secondary forest; *P.sagei*, *P.pieli*, *Cardiocondylawroughtonii*, *Recurvidrisnuwa*, *Tapinnomamelanocephalum*, *Monomoriumpharaonis* and *M.orientale* were the common species in plantations; and *Iridomyrmexanceps* and *Cardiocondylanuda* were the common species in managed farms. Ants had medium eye diameters, narrow distances between eyes, medium leg lengths and smaller body sizes in greatly-disturbed habitats; and ants had an increasing eye diameter and narrowing of the space between eyes, while the leg length and Weber’s length became shorter in moderately-disturbed habitats. Ant trait composition, based on Weber’s length, showed significantly differences amongst five habitats. The fourth corner analysis indicated that ant species traits were significantly correlated with environmental variables. The functional diversity of secondary forest, lac plantation and lac plantation-corn agroforest were higher than those in dryland farm and rice paddy. Functional diversities were significantly negatively correlated with bare ground cover and significantly positively correlated with leaf-litter cover, leaf-litter thickness and plant cover.

**Main conclusion**: Our results indicated that ant traits distribution patterns were affected by land-use changes, followed by anthropogenic disturbance pressures at the local scale. Ant traits compositions in greatly-disturbed habitats also differed from the habitats with less disturbance. It is unfavourable for the survival of the large body-size ants in more open habitats with more anthropogenic disturbance. Compared with secondary forest, dryland farm and rice paddies were less resistant and more vulnerable and lac plantations had approximately functional diversity of ant communities, suggesting that lac plantations might be resistant as secondary forest to species loss.

## Introduction

The Anthropocene is having a devastating impact on global biodiversity as the rates of human-induced climate change and land use changes are currently accelerating (Ceballos et al. 2015, Newbold et al. 2015, Newbold et al. 2016). Human-driven land use changes are recognised as the primary driver of biodiversity loss worldwide (Flynn et al. 2009, Tittensor et al. 2014, Newbold et al. 2016, Salas-López et al. 2018, Jones et al. 2018). These activities have resulted in anthropogenic interference with natural habitat loss and reduced arthropod diversity, negatively affecting ecosystem functions (Leal et al. 2012, Newbold et al. 2015, Mumme et al. 2015, Li et al. 2017). Effectively evaluating and managing different types of land is particularly important from the viewpoint of sustainable human access to resources and securing the safety and health of ecosystems (review in Kremen and Merenlender 2018). In general, arthropods with large body size species are much more affected by disturbance than small-bodied species (Gibb et al. 2018). Increased temperatures reduced the ant community body size in open habitats, while in relatively closed secondary forests, the effects of high temperatures was much less significant (Lee et al. 2021). More attention should be paid to the effects of land-use and habitat disturbance on community composition and species traits within communities in the context of climate change (Gibb et al. 2018, Salas-López et al. 2018). In the context of biodiversity loss, there is an urgent need to understand the mechanisms of species adaptability to different habitats (Kaspari and Weiser 1999, Gibb and Parr 2010, Salas-López et al. 2018).

Insect abundance and diversity declines, documented in the last century, are associated with land-use change and intensification (Hallmann et al. 2017, Seibold et al. 2019). Most of the literature exploring the relationship between human-induced disturbance and biodiversity loss have focused on taxonomic diversity. However, the information provided by species morphology taxonomy does not fully reflect the effects of human activities on biodiversity and arthropod ecological niche-based processes (Wong et al. 2019). Even without considering the interactions between organisms, discussing how environmental filtering shapes the patterns of species co-existence in insect communities and the underlying mechanisms are still a great challenge (Kraft et al. 2015). Under high-intensity land-use pressure, species are subject to significant ecological niche filtering and species tend to be small-bodied (Wong et al. 2019); however, complex landscape structures may buffer the effects of land-use pressure (Salas-López et al. 2018). To date, trait-based studies have revealed that species communities will predict functional responses to land-use intensity and climate change (Williams et al. 2010, Gibb et al. 2017). Accumulated studies have revealed that species traits are closely related to ecosystem functions and indicate changes in ecosystem function (Díaz and Cabido 2001, Díaz et al. 2003, Bernhardt-Römermann et al. 2011, Roscher et al. 2012). The trait-based approach of organisms is critical for understanding species responses to environmental change (McGill et al. 2006, Violle et al. 2007) and ecosystem functioning can be influenced by trait-based functional diversity, which reflects community assembly processes (Taudiere and Violle 2016). There is a long history in ecology using functional traits explaining how biodiversity varies in time and space. It is difficult to understand ecological processes through species-rich arthropods since their taxonomy and ecology have not been well studied (Parr et al. 2017). Ants are widely distributed in different terrestrial ecosystems and they play several important ecosystem functions, such as organic matter decomposition, soil perturbation, improvement and fertility enhancement and seed dispersal (Hölldobler and Wilson 1990, Lach et al. 2010, Hoffmann 2010). As an important component of ecosystems, ant community composition can respond to habitat disturbance. Ant species richness, abundance and composition structure could reflect changes in land-use intensities due to their sensitivity to habitat disturbance (Hoffmann 2010, Andersen 2019). The diversity reduction is related to the disturbance level (Philpott et al. 2009a) and depends mainly on the frequency, intensity and persistence of the interference, as well as the distance of the surrounding shelters (natural habitats or habitats with less disturbance) (Philpott et al. 2009b). Microhabitat conditions following habitat change affect ant community assembly (Philpott et al. 2009a, Salas-López et al. 2018). Heterogeneous and less disturbed habitats supported more species (Philpott and Armbrecht 2006, Nooten et al. 2019).

Meanwhile, with increasing focus on functional traits in ecology, ant traits are well-defined and body size and trophic groups have been commonly considered by ecologists (Parr et al. 2017). Certain traits influence species performance and fitness in a given habitat and traits determine how species can colonise and survive in given habitats, determine the strength of their interactions with other species (Salas-López et al. 2018) and even their contribution to ecosystem functions (Schmitz et al. 2004, Cadotte et al. 2011). Environmental factors of habitats can filter the distribution of ant functional traits and determine functional diversity (Yates et al. 2014, Del Toro et al. 2015). Functional diversity can be further studied, based on the measurements of functional traits of organisms, functional trait values and their ranges can be used to predict changes in ecosystem function and functional diversity emphasises the variability of trait values (Tilman 2001). Compared to the species diversity index, the functional diversity index has a stronger correlation with ecosystem function, considering the functional role of each species in the ecosystem, rather than the species diversity treating each species in terms of presence or absence and abundance. The widely recognised functional diversity indices are functional richness index (FR_ic_, volume of the functional space occupied by the community), functional evenness index (FE_ve_, regularity of the distribution of abundance in this volume) and functional divergence index (FE_iv_, divergence in the distribution of abundance in this volume) (Villéger et al. 2008). Recent advances in statistical techniques provide new insights into the relationship between species and system functioning (Brown et al. 2014, Stoklosa et al. 2014, Gibb et al. 2015a). Trait-based approaches are valuable for revealing mechanisms of ant species substitution along with land-use and climate gradients (Arnan et al. 2014, Yates et al. 2014, Gibb et al. 2015b). Recent studies have shown that ant species have morphological, physiological, behavioural and life-history traits that are strongly linked to environmental factors (Wiescher et al. 2012, Tiede et al. 2017, Nooten et al. 2019). Environmental variables are likely the major drivers of morphological features in ant assemblages (Farji-Brener et al. 2004, Gibb and Parr 2010, Wiescher et al. 2012), including body size (Gibb and Parr 2013), eye position (Gibb and Parr 2010) and leg length (Gibb et al. 2015a). Similar to other ecological traits that are linked to environmental structures (Andersen 1995), these morphological traits appear to be able to aid predictions to provide information for ecosystem management and this approach can also be used to predict large-scale patterns (Gibb and Parr 2013, Gibb et al. 2018) and an ant database will enable researchers to study ant ecology on a continental or global scale (Parr et al. 2017).

Given human needs and growing pressures on natural ecosystems, it is essential to understand how changes in land use correlate with local biodiversity changes and to explore the roles of functional traits (Barton et al. 2011, Gibb and Parr 2013, Yates et al. 2014, Gibb et al. 2015a, Tinoco et al. 2018, Brassard et al. 2020). There are apparent differences in the responses of different species to environmental stress (Andersen 1995, Hoffmann and Andersen 2003, Gibb et al. 2018). Habitat disturbances lead to smaller body szie of local ants (Lomolino 2005) and across a variety of climates, habitat disturbance selects against both small and large species (Gibb et al. 2018). The conversion of forests to agroforestry ecosystems is accompanied by a decline in species richness for many taxa (Philpott et al. 2008, Newbold et al. 2015, Rabello et al. 2021) and species composition becomes homogeneous (De la Mora et al. 2013). A reduction of forest cover by deforestation tends to make ants smaller because of the increase in microclimate temperatures (Lee et al. 2021). Further study is needed on the effects of disturbance and vegetation change accompanying land-use change on ant communities and their traits distribution.

The landscape of the south-western mountainous region of China is complex, with many different habitats. In this region, secondary forests of many ages, mixed plantations and various agricultural systems have resulted in a complex and mosaic landscape (Chen et al. 2010). In this region, where the available arable land is inadequate, it is important to meet human subsistence needs, while, at the same time, preserving biodiversity. Moreover, with climate change and increasing human-mediated disturbances, it will be important to protect habitats with complex mosaics since organisms have more options for movement and dispersal (Raven and Wagner 2021). Thus, we investigate how do habitat disturbances affect ground-dwelling ant community composition, traits distribution and functional diverity under land-use changes. Can ant functional diversity indices, such as FR_ic_, RaoQ, and FE_ve_, respond to disturbance at the local scale?

## Material and methods

**Study areas**

The study was conducted in Lüchun County, Yunnan Province, China. The region is characterised by a subtropical monsoon climate, with a mean annual precipitation of 1558 mm, an average temperature of 18.6°C, a mean warmest monthly temperature of 22.5°C and a mean coldest monthly temperature of 12.7°C over the last three decades (data from ClimateAP v.2.30, 1981-2010) (Wang et al. 2017, Nooten et al. 2019).

**Figure 1.**
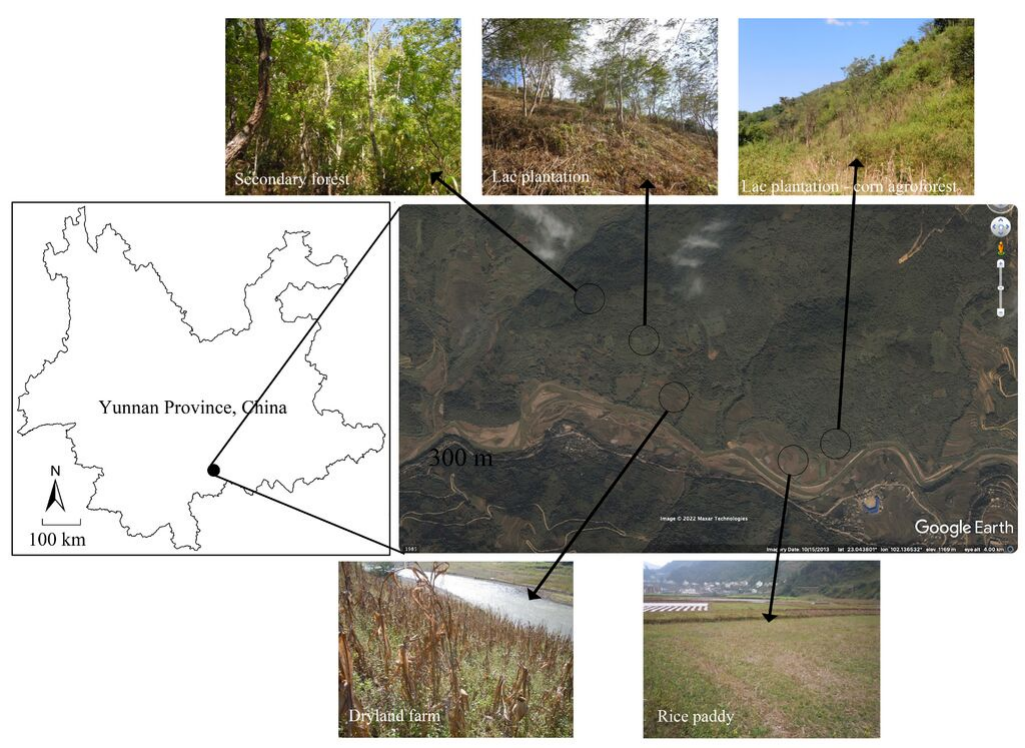
Map of Yunnan Province, China, showing the sampling sites of secondary natural forest (NF), lac plantation - corn agroforest (MP), lac plantation (LP), dryland farm (DF) and rice paddy (RP).

Five habitats were selected to investigate ant community diversity, including secondary forest, lac plantation - corn agroforest, lac plantation, dryland farm and rice paddy (Table 1, Fig. 1). Habitat disturbances caused by human production activities and, according to the distance from the place of residence and the frequency of agricultural production activities, disturbance levels are classified as high, medium and low. Six sampling sites were selected for each habitat and their plant composition, structure and environmental conditions of these sites were similar. A total of 30 sampling sites were selected, each site with an area of approximately one hectare.

Lac cultivation is a traditional agricultural practice in the mountainous and semi-mountainous regions of Southeast Asia. Lac insects are scale insects that belong to Hemiptera: Kerriidae, *Kerria*. *Kerriayunnanensis* (Ou et Hong 1990) (Hemiptera: Kerriidae) is the most common species used for lac production. Lac is the resin produced by lac insects and lac insects also secrete honeydew along with lac. Lac is a natural resin that is widely used in the food, electronics and pharmaceutical industries (Chen et al. 2010). Generally, lac plantations are composed of lac insects and their host tree species, such as *Dalbergiabalansae*. Lac plantation - corn agroforests have the same tree species as lac plantations, but the density of trees is only 1/3 that on lac plantations. Corn is planted under the trees during the rainy season (May to October) each year in the agroforests. The dryland farms, used for corn production, are also farmed from May to October. The land is generally abandoned after the corn harvest and grasses become well established during the abandonment period.

**Ant sampling and identification**

Sampling was conducted during sunny and warm weather in October 2012 and May 2013. In this region, the rainy season lasts from May to October each year. Since the region has a distinct dry and rainy season and ant community is different between the two seasons, the end of the dry season and the end of the rainy season were chosen to sample ants instead of summer months. Five pitfall-traps with 10 m space were set up within a grid in the middle of each sampling site referring to Gibb et al. 2015 and the distance between two sampling sites is more than 50 m. Traps were plastic cups 8 cm in diameter, 15 cm deep and half filled with ethylene glycol as a preservative. The traps were operated for 48 hours.

Before identification, ants were inspected in the Petri dish and then voucher specimens were point-mounted for identification using Wu and Wang (Wu and Wang 1995) and Xu (Xu 2002). Unidentified species were given a species code that was applied to this study only. Voucher specimens were deposited in laboratory of the Institute of Highland Forest Science, Chinese Academy of Forestry, Kunming, China.

**Environmental variables**

We used five quadrats (1 m × 1 m) to quantify environmental variables on each sampling site. Each quadrat was randomly placed 1 m from each trap used to investigate the environmental variables. The environmental variables were assessed visually by a square tool (the area of the square is 5% of 1 m^2^ and the area with the small square in the middle is 1% of 1 m^2^) and were a proportion of the bare ground cover (%), proportion of leaf litter cover (%) and proportion of canopy plant projection cover (%). The leaf litter thickness was calculated by the average value of 5 points within quadrat, mm).

**Morphological trait measurements**

Up to five individuals of each species were measured morphologically for functional traits. For polymorphic species, only minor workers were measured (Bihn et al. 2010, Gibb and Parr 2010). We quantified the standard linear measurements of head width, eye diameter, the distance between eyes (eye position), femur length of the hind leg and Weber’s length (Table 2, Suppl. material 1). All measurements were log (x+1) transformed before analysis (Retana et al. 2015).

**Data analysis**

The five pitfall samplings on each site for two seasons were combined to provide the statistical sample, giving n = 6 for each habitat. The abundance of each ant species of each trap was scored using a 6-point scale (1 = 1 ant; 2 = 2-5 ants; 3 = 6-10 ants; 4 = 11-20 ants; 5 = 21-50 ants; 6 = > 50 ants) to avoid data distortions from numerous ants from a single colony falling into a few traps. A shade plot was used to partition the ant assemblages, based on ant abundance (square-root transformed) using PRIMER v.7 (Clarke and Gorley 2015).

Ant Weber’s length data were square-root transformed and the number of individuals of each species in each site was used to multiply the trait value to obtain the total trait value. Bootstrap averages for the ant total Weber’s length data in all land-use types were calculated to provide means and ellipses shown in a non-metric multi-dimensional scaling (n-MDS) ordination, based on Bray-Curtis distance. PRIMER v.7 was used for these statistical analyses.

In this study, we focused on total effects of traits of all ant species amongst five habitat types instead of the difference between ant species, so we explored the distribution pattern of ant traits amongst different habitats by the kernel density plot of ant traits, using raw traits data after log(x+1) transformed, then the fourth corner analysis was applied for a fair comparison, based on the residuals of the regression of femur length, eye diameter and eye position on Weber's length, which narrow the difference of traits due to ant species. The selected traits of ant species indicated ecological functions well and different traits had different relationships with different habitat factors which we wanted to explore. Thus, we prefer to use more traits compared to some researchers. Four ant traits (eye diameter, distance between eyes, femur length of the hind-leg and Weber’s length) were utilised to construct a kernel density plot of ant species trait means amongst different sites to explore how ant traits varied amongst the habitats. Residuals of the regression of femur length, eye diameter and eye position on Weber's length were calculated using software R language, respectively. Then, we used the fourth corner method (traitglm) to analyse the relationships amongst ant assemblage compositions, species traits and environmental variables, with 999 permutations, using the “mvabund” package in the R programme (Wang et al. 2012).

We calculated FR_ic_ (the amount of functional space filled by the community), RaoQ (the quadratic entropy of Rao that incorporates both the relative abundances of species and a measure of the pairwise functional differences between species) and FE_ve_ (the evenness of abundance distribution in a functional trait space) as three complementary measures of the multivariate functional traits space and functional redundancy of different habitats using the “FD” R-package. Then, these functional trait indices and species richness amongst different habitats were compared using the Kruskal-Wallis rank-sum test, these being performed by the genescloud tools, a free online platform for data analysis (https://www.genescloud.cn). Correlation analysis amongst ant species richness, functional traits indicies and environmental variables with 95% confidence interval were conducted by software JASP 0.16.

## Results

**Figure 2.**
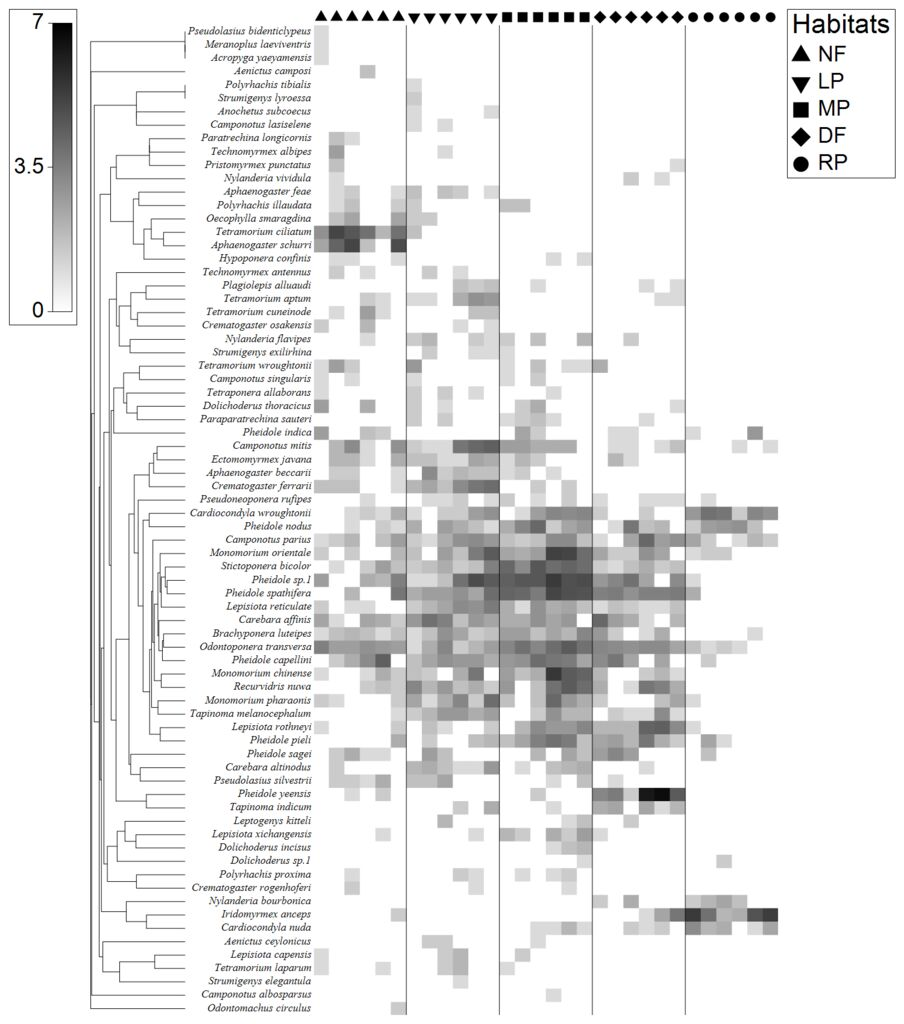
Shade plot of relative abundance of 74 ant species from 10 sites. Gradation of grey colour indicates relative ant abundance. Secondary forest (NF), lac plantation (LP), lac plantation - corn agroforest (MP), dryland farm (DF) and rice paddy (RP). Left dendrogram—74 ant species which have the strongest influence on the similarity between the samples.

We collected 14258 ants, representing 89 species, 40 genera and seven subfamilies. Of these, only 74 species could be used for trait measurements (at least five individuals). Compared with secondary forests, ant community compositions of plantations were clearly changed (Fig. 2). *Aphaenogasterschurri* and *Tetramoriumciliatum* were common in secondary forests, which have the lowest habitat disturbance. *Pheidole* sp.1, *P.sagei*, *P.pieli*, *Cardiocondylawroughtonii*, *Recurvidrisnuwa*, *Tapinnomamelanocephalum*, *Monomoriumpharaonis* and *M.orientale* were common in the plantations (lac plantations and lac plantation-corn agroforests), which had low to medium habitat disturbance. *Iridomyrmexanceps* and *Cardiocondylanuda* were also common in managed farms (dryland farms and rice paddies), which had the highest habitat disturbance.

**Figure 3.**
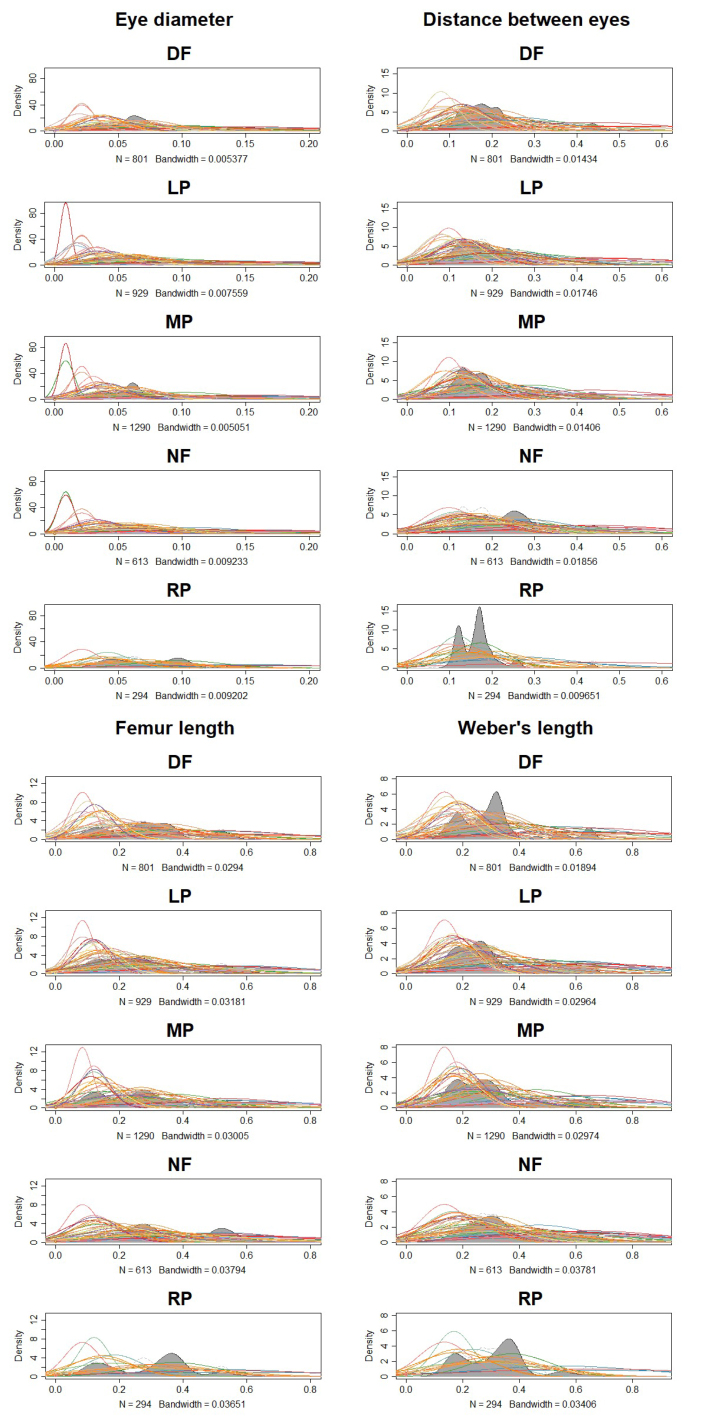
Kernel density plot of ant mean species’ traits amongst different sites. Graph created with plotDistri function from cati packages of R, which computes kernel density estimates. Secondary forest (NF), lac plantation (LP), lac plantation - corn agroforest (MP), dryland farm (DF) and rice paddy (RP). Grey contour lines represent trait kernel density and every coloured line represents an ant species.

Four ant traits (eye diameter, the distance between eyes, femur length and Weber’s length) presented different distribution patterns amongst five habitats (Fig. 3). Ant communities of secondary forests had an averaged eye diameter distribution; and ant communities of plantations (lac plantation, lac plantation-corn agroforests) and dryland farms had a medium ant eye diameter. In comparison with the above-mentioned results, ant communities of rice paddies had a relative larger eye diameter. Ant communities of secondary forest had a wider distance between eyes and significant different from those in rice paddies. In comparison to both habitats, ant communities of plantations and dryland farms had relative narrow distances between eyes. Ant communities of secondary forests also had a relatively longer leg length and significantly different from those in rice paddies. Ant communities of plantations and dryland farms had a relative averaged leg length. Ant communities of secondary forests had a medium size body and rice paddies had relatively small body size. Ant communities of plantations had more individuals with medium to small body size and dryland farms had more individuals with medium body size.

Non-metric multi-dimensional scaling (n-MDS) of Bray-Curtis distances, based on ant Weber’s length were significantly different amongst different habitats (ANOSIM global R = 0.739, p < 0.01, Fig. 4a). Ant communities of rice paddies were different from other habitats; ant communities of plantations and dryland farms were different from secondary forest. This finding is further corroborated by bootstrap averaging which shows group centroids to be reliably different of Weber’s length composition amongst different habitats (Fig. 4b).

**Figure 5.**
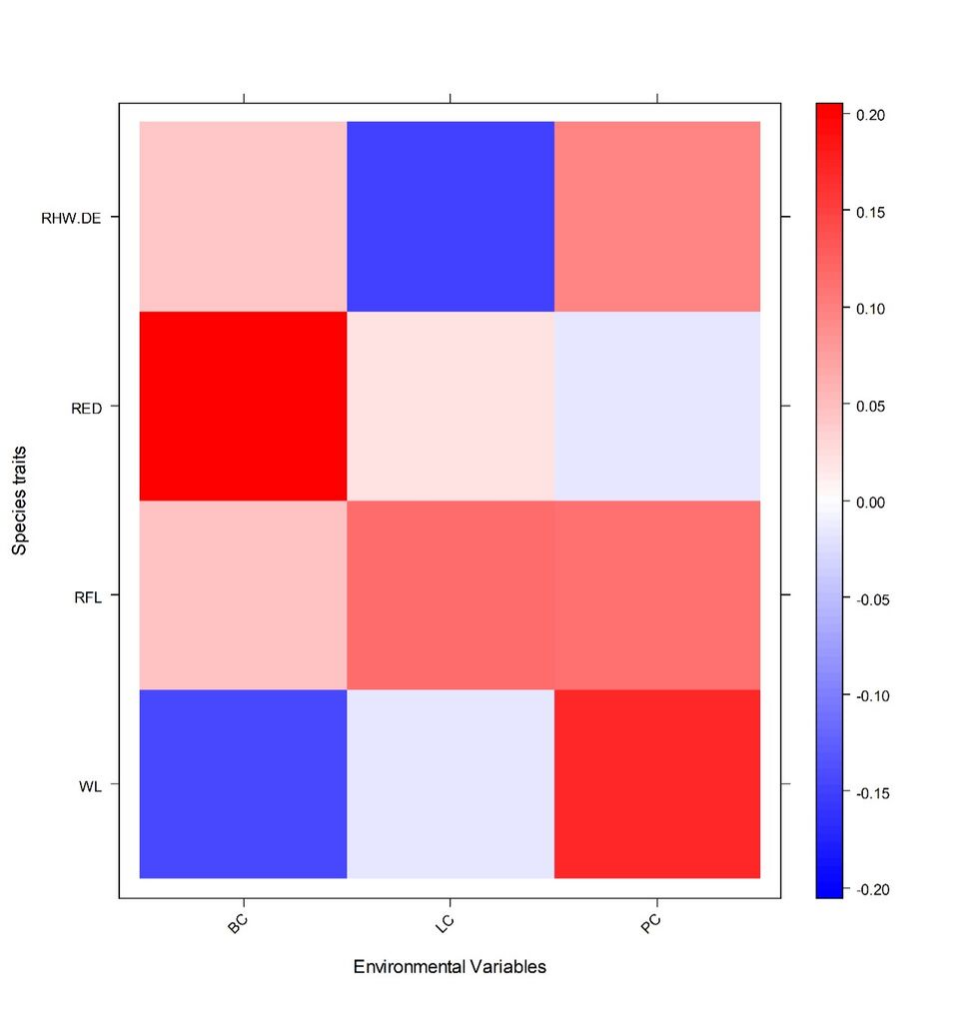
Fourth corner analysis of correlations between ant species traits and environmental factors. Significant associations are shown in blue or red, while the relative tone of colour indicates the strength of associations. Red represents a positive association and blue represents a negative association. Actual measurements for the ant species traits are Weber's length (WL), residual between femur length and Weber's length (RFL), residual between eye diameter and Weber's length (RED), RHW DE: residual between eye position (head width minus the distance between the eyes) and Weber's length (coefficients of remnants indicate what the mean abundance of traits are in comparison with pastures). BC: bare ground cover; LC: Leaf-litter cover; PC: plant cover.

Species traits were significantly correlated with environmental variables (Fourth corner analysis: p < 0.001). Ant Weber’s length was negatively correlated with bare ground cover and positively correlated with plant cover. Ant femur length was positively correlated with bare ground cover, leaf litter cover and plant cover. Ant eye diameter was positively correlated with bare ground cover. The distance between ant eyes was positively correlated with bare ground cover and plant cover and negatively correlated with leaf litter cover (Fig. 5).

**Figure 6.**
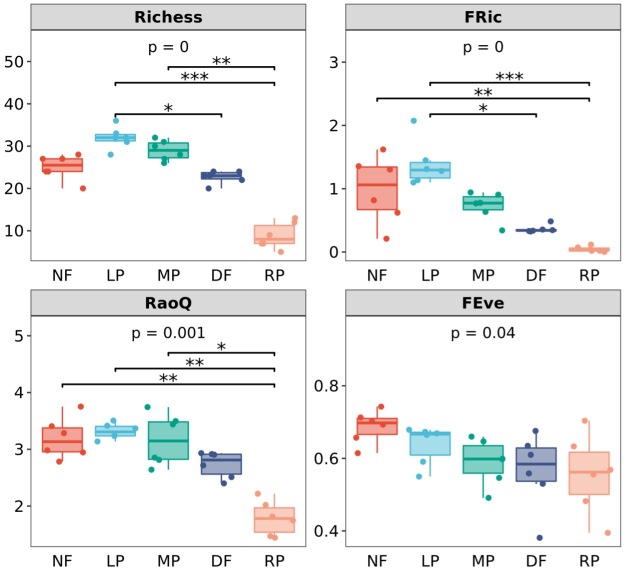
Comparison of ant functional traits indices amongst different habitats. Functional traits calculated from four ant morphological traits eye diameter, distance between eyes, femur length of the hind-leg and Weber’s length. Secondary forest (NF), lac plantation-corn agroforest (MP), lac plantation (LP), dryland farm (DF) and rice paddy (RP).

Compared with secondary forests, dryland farms and rice paddies had a significantly decreased taxonomic and functional diversity; Lac plantations and lac plantation - corn agroforests had higher species richness and functional diversity; but in the values of FE_ve_, no significant differences were observed (Fig. 6).

**Figure 7.**
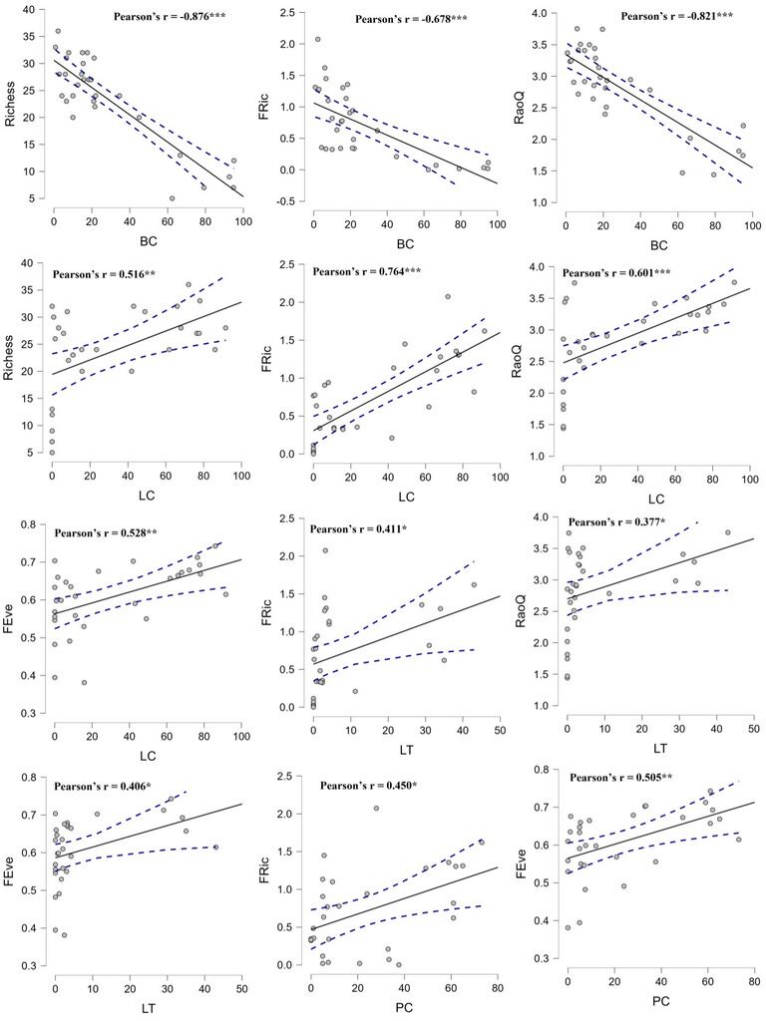
Correlation analysis between ant species richness, functional traits indices and environmental variables. * means α = 0.05 level, ** means α = 0.01 level, *** means α < 0.01 level. The dotted-dashed line indicates 95% confidence interval. BC: bare ground cover; LC: leaf-litter cover; LT: leaf-litter thickness; PC: plant cover.

Correlation analyses revealed significant associations between environments in their taxonomic and functional diversity (Fig. 7). Species richness, FR_ic_ and RaoQ indices of ants were negatively correlated with bare ground cover. Species richness, FR_ic_, RaoQ and FE_ve_ indices were positively correlated with leaf-litter cover; FR_ic_, RaoQ and FE_ve_ indices were positively correlated with leaf-litter thickness; FR_ic_ and FE_ve_ indices were positively correlated with plant cover.

## Discussion

At present, China has established many protected areas to protect biodiversity. Despite this, the role of reserves is still weak under this huge land area. Off-reserve conservation is still very important, especially low-disturbance plantations, woodlands and even agricultural lands can provide habitats for many species. We found that ant community compositions in the medium- to low-disturbance farm habitats (dryland farms, lac plantations and lac plantation-corn agroforests) were not so different from those in the secondary forests (Fig. 2). This likely occurred because the excretion of honeydew by lac insects in the low-disturbance lac plantations and lac plantation-corn agroforests could provide abundant food resources for ants, maintaining ant community diversity. Not surprisingly, numerous species showed strong affiliations with habitats and they were mostly predictable. Similar to *Aphaenogasterschurri* and *Tetramoriumciliatum*, species associated with secondary forests have been reported to occur predominantly in habitats with low disturbances (Zhang et al. 2005, Chen et al. 2011). In plantations, generalist species, such as *Pheidolecapellini*, *Stictoponerabicolor* and *Odontoponeratransversa*, are known to thrive in disturbed habitats (Chen et al. 2011) and *Cardiocondylanuda* and *Nylanderiabourbonica* are tramp species that are very tolerant to disturbances (McGlynn 1999).

Mixed agroecosystems are thought to potentially reduce the loss of biodiversity and ecosystem function due to the increased heterogeneity of their microhabitats (Perfecto et al. 2005), providing a more resilient community diversity and structure. In this study, rice paddies cannot support rich-species ant communities due to simple habitat and frequent human agricultural activity, such as irrigating, spraying, ploughing and harvesting. Compared with rice paddies, the less disturbed artificial plantations and their complex habitats provide shelter for ants. According to our previous studies, lac plantations and lac plantation-corn mixed agroforestry systems can be considered a compromise between ant diversity conservation and agricultural production in south-western China, especially, in iterms of the habitats and food resources provided for ants (Chen et al. 2010, Chen et al. 2011). Firstly, as part of the lac production system, host plants of lac insects and shrubs created complex habitats for ants, while the honeydew secreted by the lac insects provided rich food source for ants (Chen et al. 2011). Therefore, it can be inferred that this lac-agricultrual system is an important model for the conservation of ant diversity and will improve the performance and resilience of ecosystem services in this region.

The trait which strongly influences its organism performance is termed a functional trait (McGill et al. 2006). Habitat filtering ultimately changes the distribution patterns of features within communities, promoting ecological niche differentiation and increasing phenotypic variation amongst species (Taudiere and Violle 2016). Our study found that ant trait distribution patterns differed amongst the five habitats. Ants in more disturbed and open habitats had relatively larger eyes, forward vision, shorter legs and smaller body size, whereas the backward vision, wide field of view, fast-moving (long leg length) ants were found in complex habitats, such as secondary forest. This is slightly different from the study of Gibb (Gibb et al. 2018), who found both the largest and smallest species were absent from disturbed ecosystems and natural habitats showed heterogeneous ant trait distribution patterns. Gradients of land-use intensity can have a filtering effect on species, screening out smaller-bodied, highly dispersed taxa and reducing specialised species (Wong et al. 2019). For example, ants in simple habitats usually have longer legs (Gibb and Parr 2013). In the more open and less complex habitats, the legs are longer because ant movements are limited in a complex habitat (Gibb and Parr 2010, Arnan et al. 2014, Retana et al. 2015). Additionally, ant eyes tend to be larger, influencing their vision (Gibb and Parr 2013). Ant species whose body size falls towards either the largest or smallest extreme are more susceptible to habitat disturbance, with disturbance resulting in the loss of large and small predators, homogenising body size distribution (Gibb et al. 2018). Our finding was different from former reports of several morphological traits of ant assemblages responding to changes in habitat complexities driven by disturbance. In our study, at a local scale, after filtering by habitat disturbance, the traits of ant species amongst the land plots correlated with the environmental variables. First, ant body size was negatively correlated with bare ground cover and leaf litter cover; in more open and simple habitats, ants had larger body size, medium eye size, forward vision and medium leg length; we assumed that larger ants may not go through a narrow gap in leaf litter and have a high risk of predation. Second, the ant femur length was positively correlated with leaf litter cover and plant cover. This may be because ants had a stronger climbing ability in complex habitats with plants and leaf litter cover and this finding is not supported by the relative increase in leg length with decreasing ground cover (Wiescher et al. 2012). Third, ant eye size was positively correlated with bare ground cover; it seems that ants had good visual performance in habitats with barer ground cover. Fourth, the distance between eyes was positively associated with plant cover; we assumed that ants had more lateral vision in the forest than in the open habitat.

Species richness and abundance are the basis of ecosystem function, while the realisation of ecological function depends on the functional traits possessed by species (Tilman 2001). Functional traits determine the species response to environment disturbance (Mouillot et al. 2013). Thus, ecosystem function is not determined by the number of species, but by the functional traits that species present (Hooper and Vitousek 1997). High functional diversity enables more efficient ecological functions and increases productivity and resilience (Mason et al. 2003), while overlapping ecological niches also increase redundancy of ecological functions and improve ecosystem stability (Song et al. 2014). In this study, compared to natural habitats, agricultural habitats had a negative effect on the species richness and functional diversity of ant communities. More than reducing the amount of functional traits space occupied by ant communities (FR_ic_), dryland farms and rice paddies also decreased the functional redundancy (RaoQ). Plantations had over half of the ants species that were found in the secondary forests. They had approximately the amount of functional traits space occupied by ant communities and functional redundancy, suggesting that the functional diversity of ant communities in plantations might be resistant to species loss as natural habitats. Ant communities in agricultural habitats with high disturbance were less resistant and more vulnerable. Whereas plantations with low disturbance had higher similarity amongst ant species, resulting in a functional homogenisation of ant communities.

Some studies have shown that ant traits are not associated with environmental variables at the local scale (Yates et al. 2014), but we found that ant body size, eye size, eye position and leg length were associated with environmental variables in our selected study sites within 5 km. Here, we conclude that environmental variables filter ant traits at a local scale, based on morphological measurements. Other ant traits, such as life history, ecological traits, colour and pilosity, should be investigated in the future. In this study, we used the mean value of traits of species for all analyses. Considering intraspecies variation and intercommunity variation, morphospace-, ecological niche- and individual-based trait variation need to be considered for more in-depth studies in the context of land-use changes.

## Supplementary Material

B7D0786B-350F-554B-8706-A4BE1126A807 10.3897/BDJ.10.e85119.suppl1 Supplementary material 1 Morphological traits data of ants Data type morphological File: oo_709091.xlsx https://binary.pensoft.net/file/709091 Xiang Zhang

## Figures and Tables

**Figure 4a.**
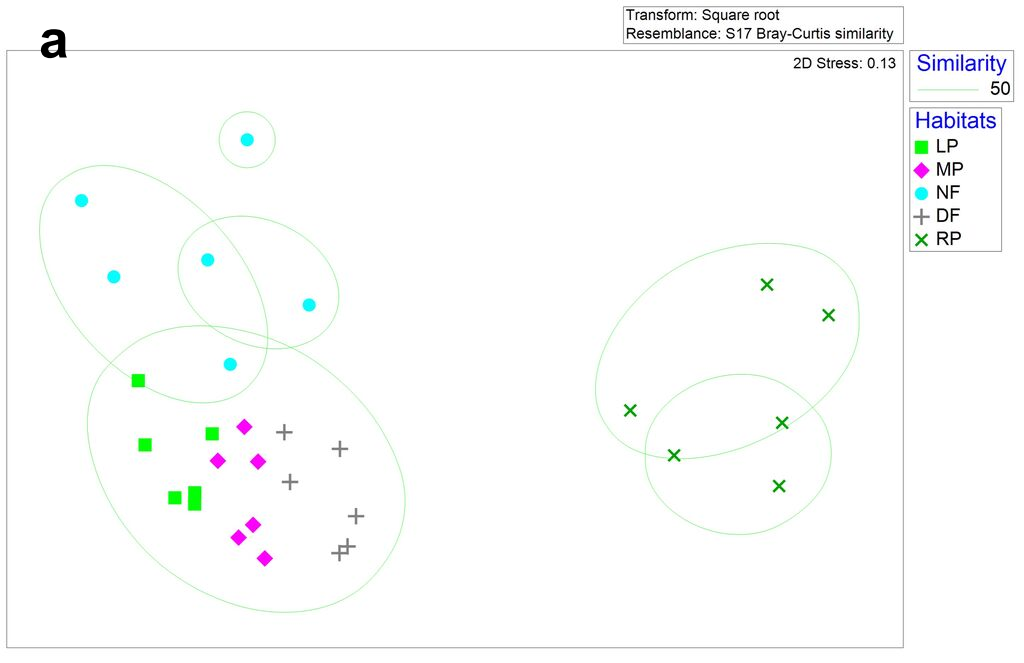


**Figure 4b.**
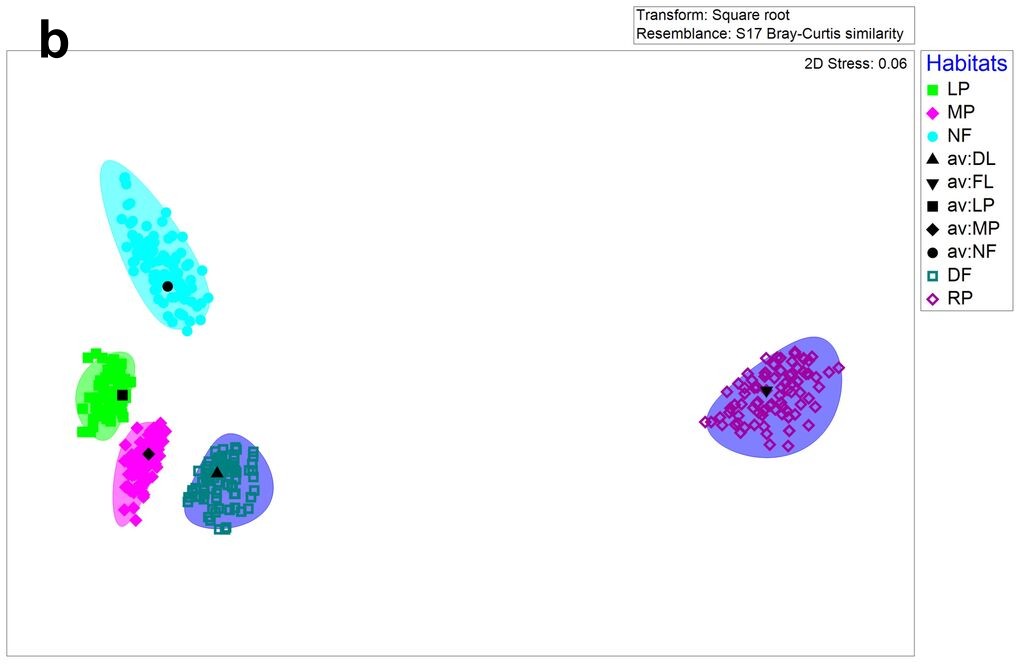


**Table 1.** Information of sampling sites.

Habitats	Altitude (m)	Canopy density %	Average bare ground cover %	Average stone cover %	Average leaf litter cover %	Average leaf litter thickness (mm)	Average plant cover %	Habitat disturbances caused by human agricultural activities	Characteristics
NF	1158~1235	68.7~75.0	13.4~29.9	0.5~3.1	63.3~81.9	25.7~35.3	51.7~64.7	Low	Evergreen broad-leaved forest with shrubs and thick leaf litter, fewer grasses. Very few humans enter and there is basically no disturbance.
LP	1021~1065	52.7~70.0	1.9~10.7	0.7~1.3	52.7~72.7	3.2~3.8	6.6~47.4	Low to medium	Six-year-old lac insect hosting tree species such as *Dalbergiabalansae*, diverse grass species. About 4 weeks of the year are devoted to harvesting lac and rearing lac insect.
MP	983~997	39.0~39.3	15.7~17.1	1.5~4.3	2.5~4.1	0.2~0.8	5.8~14.5	Medium	Six-year-old lac insect hosting tree species such as *Dalbergiabalansae*, but about 1/3 density compared with lac plantation. Corn was planted between the trees from May to October. Lac production was simultaneous with corn-producing. About 4 weeks of the year are devoted to harvesting lac and breeding lac insect, while about six months are devoted to producing corn.
DF	997~999	-	6.9~21.5	0.1~10.3	11.7~16.7	1.8~2.3	0.3~0.4	Medium to high	Corn production is conducted from May to October, after which the land is left fallow. During the fallow period, the grasses become well developed.
RP	967~1030	-	69.4~94.1	-	-	-	5.8~30.6	High	Rice paddy with hard soil after harvesting. Some low grasses present. Vegetables are grown after harvesting rice and it is used for agricultural production for more than 8 months of the year.

**Table 2.** Description of ant traits related to functions

Ant traits	Code	Expected functions	References
Head width	HW	Pass through microhabitat	Kaspari and Weiser (1999)
Eye diameter	ED	Visual	Weiser and Kaspari (2006)
Distance between eyes	DE	Visual, microhabitat	Silva and Brandão (2010)
Femur length of the hind leg	FL	Foraging speed	Kaspari and Weiser (1999)
Weber’s length	WL	Body size and resistance	Weber (1938)
